# Supplementary figures and images for: Optogenetic and chemical genetic tools for rapid repositioning of vimentin intermediate filaments
Source: J Cell Biol. 2025 Jul 8;224(9):e202504004. doi: 10.1083/jcb.202504004 (PMC12237251; doi:10.1083/jcb.202504004)

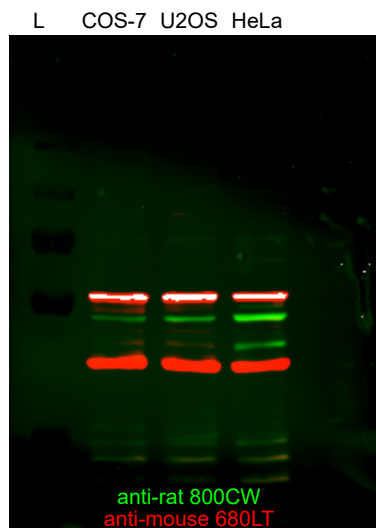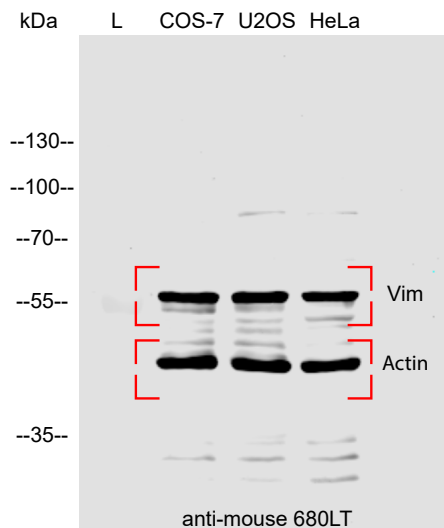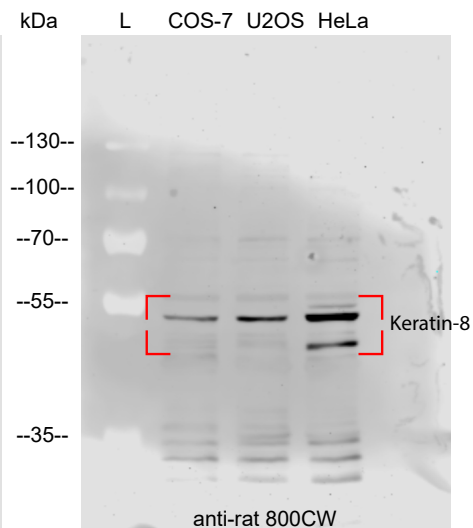

Supplement: SourceData FS4 — is the source file for Fig. S4. [file jcb_202504004_sourcedatafs4.pdf]
